# Validity of mother-child self-perceived oral health for the assessment of 5 years old children’s oral health in Indonesia

**DOI:** 10.1186/s12903-023-02876-5

**Published:** 2023-03-25

**Authors:** Safira Khairinisa, Febriana Setiawati, Diah Ayu Maharani, Risqa Rina Darwita

**Affiliations:** Department of Preventive and Public Health Dentistry, Faculty of Dentistry, Universitas Indonesia, 10430 Jakarta, Indonesia

**Keywords:** Self-perception, Child, Mother, Oral health, Early Childhood Caries

## Abstract

**Background:**

Early childhood caries (ECC) is a serious condition that has a negative impact on young children’s quality of life. Mothers’ perceived need for oral health care plays an important role in their children’s oral health behavior. This study aimed to compare mother and child self-perceived and dentist-evaluated needs for oral health care.

**Methods:**

This cross-sectional study included 266 preschool children aged 5 years old and their mothers. A self-administered questionnaire to the mothers and interviews with the children were used to assess the perceived needs of oral health care. The mothers were asked to rate their children’s oral health and determine if they needed dental treatment. The children were also asked how they felt about their oral health and whether they had any tooth decay. Agreement between mother and child regarding the child’s oral health was assessed. The evaluated needs were assessed clinically using the **dmft** (decayed, missing, and filled teeth [primary dentition]) and **pufa** (pulpal involvement, ulceration, fistula, and abscess [primary dentition]) indices. The perceived and evaluated needs were compared using spearman analysis to determine their correlations and the validity of the perceived needs compared to the clinical examination was assessed using the area under the curve (AUC), sensitivity (Sn), specificity (Sp), and likelihood ratio (LR).

**Results:**

The prevalence rate of ECC was 89.4%, with 35% having at least one condition from untreated caries (pufa > 0). Mothers and children have a fair agreement regarding the child’s oral health (ICC = 0.335). When comparisons were conducted between perceived and evaluated conditions, Mother’s rating about their child’s oral health showed the strongest correlation to dmft index (*r* = 0.372; p < 0.001). Several accuracy parameters done in this study (AUC, Sn, and Sp) did not meet the acceptable threshold. The sensitivity and specificity were the highest when comparing mothers’ perceived need for their child’s dental treatment to the dmft index (Sn = 96.7%) and pufa index (Sp = 88.1%), respectively.

**Conclusion:**

Compared to the dentist’s assessment, the mother and child self-reported oral health statuses showed lower accuracy in assessing the child’s condition. But, the mothers in this study were better than their 5-year-old children at perceiving their child’s oral health care needs. As a result, these subjective assessments can be used as a complement, but not as a substitute, to the actual clinical evaluation.

## Background

Dental caries is the most prevalent oral health problem, affecting a large number of people worldwide [1]. Early childhood caries (ECC) is defined as the presence of one or more teeth with cavities, missing, or restored because of caries in children younger than 72 months. This oral health problem is a global health burden, medically, socially, and economically [1, 2]. Indonesian national oral health insurance allows all Indonesian to access healthcare [3]. But, Indonesia has the highest prevalence of ECC among several other countries, with 90% in the 5-year-old children population [4]. Jakarta, as the capital city of Indonesia, is different from other provinces, where there is a lack of remote population and different gross domestic product and healthcare structure [5]. According to a spatial analysis in 2021, Jakarta has the most dentist in each public health center work area in Indonesia, along with the unevenly distributed private Indonesian dentists are centralized in this city [6, 7]. Even though it still has several limitations, inequality analysis showed that Jakarta had one of the lowest levels of health inequality in Indonesia (3.1% compared to 92.3% in the Province of Papua) [8, 9]. So, access to oral healthcare services in Jakarta is relatively adequate compared with other parts of the country. Yet, the prevalence of oral health problems in the city is still high [10].

ECC is a serious condition that has a negative impact on young children’s quality of life. Their primary caregivers, usually their mothers, play a crucial role in taking care of their health [11, 12]. Mothers play an important role in providing effective guidance and positive attitudes toward oral health [11]. In five years old children, the primary dentition represent the late stage and can be used to evaluate the efficacy of previous oral healthcare behavior. Identifying problems at this stage may help to prevent future oral health problems [13].

Health needs are defined as the degree of health disease that potential users of healthcare experience [14]. Along with the recognition of health as a subjective state, the self-perceived need for oral health care is just as important [15]. A perceived need that is different from the actual oral health status indicates unawareness, which must be overcome [16]. Regardless of the clinical parameters, multiple variables, such as socioeconomic factors and oral health literacy, could determine individual perceived needs [15, 17]. The perceived need for dental care and parents’ awareness of their child’s health will determine whether they seek dental care.

Aside from being a useful parameter of individual awareness, self-rated oral health status is also useful in epidemiological surveys, where clinical examinations, especially in large populations, are impractical and costly. One of the most efficient methods for obtaining oral health data in a population is through self-report, which reflects their perceptions of oral health status and the perceived need of care [15]. It is important to compare perceived and evaluated needs across populations and groups because subjective assessments may vary depending on individual beliefs and awareness, oral health, social, educational, cultural background, and environmental conditions [15, 16]. To date, no study has assessed the difference between the mother and child self-perceived and dentist-evaluated needs of 5-year-olds in Indonesia. This study aimed to compare the oral healthcare needs of children aged five years old in Jakarta, Indonesia, as perceived by mothers, children and clinically evaluated. Furthermore, the accuracy of the mother- and child-perceived oral health compared to the clinical assessment as the gold standard was analyzed.

## Methods

The reporting of this study was in accordance with the STARD (Standards for Reporting of Diagnostic Accuracy) guidelines [18]. This cross-sectional study was conducted in 7 preschools in Jakarta from August to October 2019. The preschools were chosen from districts in East Jakarta via multistage cluster random sampling. Jakarta Province was divided into six cities, which one district was selected at random: East Jakarta. East Jakarta consisted of 10 districts, and one district was chosen randomly. Seven of the 37 preschools in the chosen district were chosen at random. The local community health center assisted in the sampling and authorization process; thus, the selected school agreed to participate. Sample size estimation suggested that a total of 227 pairs of mothers and children completing the study would be sufficient for detecting statistical significance with a power of 95% and significance level of 0.05, assuming an effect size of 0.35 [19]. A total of 262 mother-child pairs who met the inclusion criteria were recruited. The inclusion criteria for participants were children aged 5 years and their mothers who were willing to participate and children with no other medical condition(s) that could confound the study outcomes.

Before the clinical examination, the mothers provided written informed consent for their and their children’s participation in this study. This study was reviewed and approved by the ethics committee of the Faculty of Dentistry, Universitas Indonesia (protocol No. 010730719). Data were obtained prospectively through self-administered questionnaires completed by the mothers, oral health examinations, of the children and child interviews.

### Mother’s questionnaire

Mothers were asked to complete a self-administered questionnaire pertaining to their demographic data (sex, date of birth, number of siblings, mother’s age, and mother’s education level) and rating their children’s oral health, with very good, good, acceptable, poor, and very poor (scale of 0–4) as possible responses. To measure agreement between the mothers’ ratings and their children, the mothers’ answers were re-classified into three categories: 0–1 as good, 2–3 as fair, and 4 as poor (scale of 0–4 into 0–2). They were also asked about their perceptions of their children’s needs for dental treatment (“Need” or “Did not need”) [20]. The level of education was categorized into low for those who completed primary or secondary school, moderate for those who attended high school, and high for those who attended university. All mothers who participated in this study were able to read, write, and give their written consent.

### Child interviews

The children were interviewed face-to-face on the same day of the clinical examination, away from their parents, to avoid their parents’ potential influences on their responses [20, 21]. The interviews were conducted by the same interviewer throughout the entire study, who had previously been trained and calibrated to conduct an interview using easily understood language for children to reduce potential bias. Children were asked to rate their oral health with a face card indicating whether they were “happy,” “fair,” or “sad” (scale of 0–2) of their oral health. They were also asked (yes/no) if they thought they had tooth decay [20].

### Clinical examination

The clinical examination of the preschool children was performed by two trained and calibrated examiners using sterilized dental mirrors, World Health Organization (WHO) probes, and intraoral light-emitting diode lights. Dental caries was diagnosed using WHO’s dmft criteria, which included recording every **d** (decayed tooth), **m** (missing tooth due to caries), and **f** (filled tooth) in the primary dentition was recorded. When there was a lesion in a pit, fissure, or smooth tooth surface, undermined enamel, an unmistakable cavity, or a detectably softened tooth surfaces, the decayed tooth was recorded as present [13]. Later, the dmft score of each 5-year-old child was classified into free caries (dmft score = 0), ECC (dmft score = 1–5), and severe ECC (dmft score ≥ 6) [22].

To assess the presence of oral health conditions resulting from untreated caries, the pufa index score was also recorded. These index diagnostic criteria are visible **p** (pulpal involvement), **u** (ulceration caused by dislocated tooth fragments), **f** (fistula), and **a** (abscess) [23]. Both the dmft and pufa index scores, ranging from 0 to 20, were calculated in the same cumulative way, representing the number of teeth that met the diagnostic criteria. For calibration, both examiners examined 20 children, and after review and discussion, a consensus was reached on each code. Interexaminer reproducibility based on the kappa value of the dmft and pufa scores was 0.93 and 0.98, respectively. Furthermore, intraexaminer reliability revealed almost perfect agreement in each index for both examiners (Kappa = 0.87–0.98).

### Statistical analysis

The chi-square test was used to test for statistical significance of observed differences outcomes (tooth decay and its untreated complications [pufa]) in different categorical variables such as sex, the number of children (single child, ½ siblings, or > 2 siblings), and mother’s education level (low, moderate, high). Spearman correlations were used to assess the strength and statistical significance of the correlation between the mother-child self-rated oral health and dmft and pufa index scores. The interclass correlation coefficient (ICC) was used to assess agreement between the mothers’ ratings and their children after reclassifying the mothers’ ratings into a 0–2 scale [24].

The diagnostic validity of self-reported tooth decay and mother-perceived need for treatment was compared to the clinical examination using sensitivity, specificity, and receiver operating characteristic (ROC) analysis [15, 16, 25]. Sensitivity refers to a screening test’s ability to detect a true positive, which is all people who do have an oral health problem, while also not categorizing other people as not having a problem when they do (false negatives) [true positives divided by (true positives plus false negatives) x 100]. Specificity, on the other hand, refers to a screening test’s ability to detect a true negative, which is all people who do not have an oral health problem, and not classifying people as having a problem when they do not (false positives) [true negatives divided by (true negatives plus false positives) x 100] [26]. The area under the curve (AUC) was used to quantify the ability to discriminate between the presence and absence of current oral health conditions [26]. The likelihood ratio (LR) was calculated to assess accuracy based on how much self-reporting increased or decreased the condition’s prereporting probability. Typically, a positive LR of ≥ 10 and a negative LR of ≤ 0.1 are considered to represent informative tests [15].

## Results

This study analyzed data from 266 pairs of mothers and children, with almost equal numbers of male (n = 134) and female (n = 132) children. Most mothers who participated in this study had 2 or 3 children and attended high school as their highest educational attainment. Intraoral examination revealed that from the total sample, the children’s mean dmft score was 7.7± 5.5 and the pufa score was 0.9±1.7. Almost all children had decayed teeth, with 28.6% (n = 76) and 60.1% (n = 160) of them having ECC and severe-ECC, respectively [22]. In addition, the pufa index showed that 35% of the children had at least one condition resulting from untreated caries [23].

Table 1 shows the mothers’ and children’s perceptions of their child’s oral health, with lower scores indicating more positive ratings. On a scale of 0 to 2, most children were happy with the condition of their teeth (0.48±0.64). More than half of them (55%; n = 147) claimed they did not have oral cavities. Most mothers thought that their child’s oral health was relatively good (1.57±1.55; on a scale of 0–4), but 68.4% believed that their child needed dental treatment.

**Table 1:** Mother-Child Self-Perceived Oral Health of Children (n = 266)

	Mean±SD	*n* (%)
**Self-Perceived Oral Health of Children**		
Self-rated oral health (0–2)	0.48±0.64	
Presence of Dental Cavity		
*Have cavities*		118 (44.4%)
*Did not Have Cavities*		147 (55%)
**Mother-Perceived Oral Health of Children**		
Mother-rated of their child’s oral health (0–4)	1.57±1.55	
Mother perceived-need of child dental treatment		
*Need Treatment*		182 (68.4%)
*Did not Need Treatment*		84 (31.6%)

**Table 2:** Sociodemographic characteristics of the study participants (n = 266) and association with clinically assessed oral conditions

Subject Characteristic (*n)*	d-t > 0 (%)	*p value* ^*a*^	pufa > 0 (%)	*p value* ^*a*^
Gender of Child				
Male (134)	120 (89.6%)	0.555	49 (36.6%)	0.580
Female (132)	121 (91.7%)		44 (33.3%)	
Number of Children				
Single (47)	42 (89.4%)	0.944	14 (29.8%)	0.675
1/2 siblings (198)	180 (90.9%)		70 (68.2%)	
>2 siblings (20)	18 (90%)		8 (40%)	
Mother’s education				
High (102)	88 (86.3%)	0.133	93 (35%)	0.752
Moderate (103)	125 (94%)		48 (36.1%)	
Low (31)	28 (90.3%)		12 (38.7%)	

a = chi-square significant at 0.05

Table 2 shows the differences between clinical assessment among sociodemographic factors based on sex, parental education level, and the number of siblings; no statistically significant differences were found among them (p > 0.05). Table 3 shows the correlation between the child oral health scores given by mothers and the children with the actual condition measured by the dmft and pufa indices. The Spearman correlation found significant correlations between perceived and evaluated variables (*r* = 0.141–0.372). When comparing both perceived variables, The ICC analysis showed only fair agreement between the mothers and their children regarding the child’s oral health (ICC = 0.335).

**Table 3:** Association between Mother-Child self rated oral health with clinically assessed oral condition in 5-year-old children (n = 266)

	dmft index		pufa index		Mother-rated of their child oral health
	*r value* ^*a*^	*p value*	*r value* ^*a*^	*p value*	*ICC* ^*b*^
Child self-rated oral health	0.187	0.002*	0.141	0.021*	0.335
Mother-rated of their child oral health	0.372	< 0.001**	0.272	< 0.001**	

a = spearman correlation analysis; *significant at 0.05; **significant at 0.01 ; b = *Interclass Correlation Coefficient* to measure agreement between child and mother

The validity of the mother- and child-perceived need was evaluated using ROC analysis (Table 4). Compared to the pufa index score, the child self-reported tooth decay had the highest AUC score (AUC = 0.67). The sensitivity of child self-reported tooth decay was higher than the specificity when compared to the presence of dental caries (dmft); however, compared to the outcomes of untreated dental caries (pufa), the specificity was higher than the sensitivity. The mothers’ perceived need for dental care had the highest sensitivity (Sn = 96.7%) to the presence of dental caries and the highest specificity (Sp = 88.1%) to pufa. Compared to the pufa index score, the mother-perceived need for dental treatment had the highest LR + of 3.83, and the child-perceived presence of tooth decay had the lowest LR − of 0.67.

**Table 4:** Validity of the mother- and child-perceived need of oral-health care compared with decay tooth and untreated dental caries complication (n = 266)

	d-t > 0						pufa > 0					
	SN (%)	SP (%)	LR+	LR-	AUC	95% CI	SN (%)	SP (%)	LR+	LR-	AUC	95% CI
Child perceived-presence of dental cavity	93.6	11.6	1.06	0.51	0.561	0.442–0.681	48.7%	76.2%	2.06	0.67	0.634	0.563–0.704
Mother perceived-need of child dental treatment	96.7	22.6	1.25	0.15	0.255	0.153–0.357	45.6%	88.1%	3.83	0.61	0.340	0.274–0.405

*SN* sensitivity, *SP* specificity, *LR +* likelihood ratio for positive value, *LR-* likelihood ratio for negative value, *AUC* area under curve

## Discussion

In this study, 5-year-old children had a high level of dental caries. At this age, parents, especially mothers as the primary caregiver, play an important role. However, inadequate awareness, low maternal oral health literacy, and the mother’s lack of locus of control are associated with children having dental caries and adverse early childhood oral health outcomes [17]. These factors may influence the perceived need for children’s dental care and whether they have a good oral health behavior.

A previous study found that the child’s sex and the mother’s education were both associated with a high prevalence of caries in children [27, 28]. Having siblings also has been linked to decreased dental visits and oral health-related quality of life [29]. But, this present study showed no statistically significant differences in clinically assessed decay and pufa between different sex, the number of siblings, or maternal education.

Self-perceived assessment is becoming more popular in oral health studies [25]. As a screening test, self-perceived need assessment is not invasive, less expensive, less time-consuming, and less discomforting for patients. Although these tests are known to be unreliable and ambiguous, the extent of their accuracy must be determined to encourage appropriate interpretation and decision-making [15, 25]. In young children, their mothers usually act as their proxies [30–32]. However, several studies have discussed children’s self-report capacity, which assumed that children as young as eight years already have mental functions for accurate self-evaluations [20]. Another study even suggested that children as young as 4–6 could accurately describe their conditions in specific domains such as dysfunction and pain [33].

Although it is widely accepted that children under the age of five cannot reliably self-report, clinical practice has considered children’s perceptions of their disease and opinions about their treatment [37]. The cut-off age for this study was set at five years old, at which children are considered capable of reporting their condition. This population already has social and emotional capacities such as communication, self-confidence, self-control, cooperation, curiosity, and intellectual skills [38]. However, we also acknowledge that 5-year-old children, even those from the same population, are not always developmentally identical [20]. A child’s characteristics and maturity are influenced by various factors, including the parents’ behavior and beliefs, which later provide an environmental framework for children’s psychosocial growth also shape their behavior and perceptions [39]. Nonetheless, it is necessary to determine how far 5-year-old children and their mothers in Indonesia can report their oral health condition.

Children and their mothers’ recognition of oral health problems may be related to the oral health-related quality of life (OHRQoL) they experienced. Pakkhesael et al. (2021), in Iran, found that parents are more concerned about their toddler’s oral health and have lower OHRQoL due to increased dmft [34]. In Indonesia, Ramadhani et al. (2021) also found that children’s and parents’ perceptions of their OHRQoL are related to the dmft and pufa scores [19]. Several studies have already been conducted to assess the perceived needs for oral health care in various age groups. In Thailand, the perceived need for dental treatment among school-aged children was related to the number of untreated decayed teeth. It was highly associated with levels of oral impacts, specifically on eating, emotional stability, and smiling performances [35]. However, Rajput et al. in India discovered that only one-third of children’s parents that dental problems are just as serious as other health problems and considered their child’s oral health was unsatisfactory [36]. In Indonesia, Maharani et al. in 2019 showed that self-perceived information provided by young adolescents cannot accurately evaluate their oral health conditions [25].

Among general population, self-report should always be used where possible. However, in young children whose age or cognitive/health status prevents them from reliably self-reporting, proxy reports are a valuable way of obtaining information about them. But, multiple studies have found inconsistencies between under and over-estimation among proxy reports [37]. Previous research has shown that parental ratings are usually worse than children’s self-ratings. Mothers are more accurate in indicating their children’s health and clinical needs, especially in observable conditions [20, 29, 33]. According to this study, mother and child only have a fair agreement regarding children’s oral health condition. So, obtaining information from both whenever possible should be encouraged to avoid the loss of information.

When compared to both mother and child perceptions of oral health needs, the pufa index showed higher specificity than sensitivity. The higher specificity (ability to identify true positives) of the pufa index may be highly related to the child’s pain as a complication of untreated caries, making both mother and child aware of the actual problem [23]. Thus, dental caries and the need for oral health care were not recognized until the lesion was already extensive and painful. At that time, the disease required a more invasive procedure [31].

On the contrary, the sensitivity (ability to identify true negatives) of mothers’ assessment was the highest compared to tooth decay’s actual presence. However, the high prevalence of dental caries among respondents may mask the false positives. The higher sensitivity of the dmft index and specificity of the pufa index were similar to a previous study in Jakarta but in a different age group (12–15 years old) [15]. Thus, compared to the dmft index, the pufa index used in the clinical assessment may have reduced the likelihood of false-positive reports. Nonetheless, the few shortcomings of the pufa index, such as closed fistulae, which are often not visible on intraoral examination, should not be overlooked [40].

The main finding of this study is that several accuracy parameters (AUC, Sn, and Sp) did not meet the acceptable threshold [15, 26]. This finding indicates that when mothers and children are asked to self-report their oral health, they do not provide accurate information. The correlations between the variables were significant but relatively weak, so they could not be used to predict the actual oral health condition [20]. However, if the pain is present, both mother and child can identify their problem more easily. This finding is similar to previous studies that found disparities between clinical and self-reported oral health among different populations and age groups [14–16, 25, 35]. This inaccuracy could be attributed to the poor understanding of oral diseases and their associated symptoms. However, this method may have practical applications in epidemiological studies and rapid screening to determine the need for referral to higher-level of health care facilities [13, 15]

As their children’s primary caregiver, mothers establish their children’s behaviors related to oral health. Mothers’ awareness of their children’s worsening oral health conditions is a key factor in seeking dental treatment [29]. Promoting oral health awareness can help a person recognize the problem and make positive health-related behavioral changes, even in individuals as young as five years old [30, 41]. Both mothers’ and children’s oral health awareness should be improved so that they have the better diagnostic ability and can implement preventive measures as early as possible, resulting in better oral health outcomes in the future.

There are several limitations to this study. The sampling method might not have produced a representative sample of children aged 5 years who did not attend school, introducing a selection bias. Second, random errors due to the sampling method could have been present. The results of this study must be inferred carefully from larger populations. Social desirability bias might have been present because of the potential embarrassment of some participants (on certain topics). They may have chosen to provide information that is suitable for their image rather than the actual condition [42]. Furthermore, other factors not observed in this study could have influenced mothers and their child’s perceived need of oral health care.

## Conclusion

This study found a statistically significant difference between the mother- and child-perceived and evaluated oral health status of 5 years old children. Regarding assessing children’s actual oral health conditions, the mother-child self-reported oral health status was not as accurate as clinical assessments, but mother’s report was slightly more accurate. As a result, these subjective assessments can be used as a complement, but not as a substitute, to the actual clinical evaluation. Improvements in oral health promotion are required to increase mothers’ and children’s oral health awareness so they can have better diagnostic ability and children can receive dental attention as early as possible.

## Data Availability

The raw data are not publicly available due to ethical restrictions but are available from the authors to any author who wishes to collaborate with us.

## Abbreviations

ECC: Early Childhood Caries
dmft: decayed, missing, and filled teeth [primary dentition]
pufa: pulpal involvement, ulceration, fistula, and abscess [primary dentition]
Sn: Sensitivity
Sp: Specificity
LR: Likelihood ratio
AUC: Area under the curve
ROC: Receiver operating characteristic
CI: Confidence interval.
